# The Impact on HIV Testing Over 6 Months When Free Oral HIV Self-Test Kits Were Available to Truck Drivers in Kenya: A Randomized Controlled Trial

**DOI:** 10.3389/fpubh.2021.635907

**Published:** 2021-09-30

**Authors:** Elizabeth A. Kelvin, Gavin George, Matthew L. Romo, Joanne E. Mantell, Eva Mwai, Eston N. Nyaga, Jacob O. Odhiambo, Kaymarlin Govender

**Affiliations:** ^1^Department of Epidemiology and Biostatistics, CUNY Graduate School of Public Health and Health Policy, City University of New York, New York, NY, United States; ^2^CUNY Institute for Implementation Science in Population Health, City University of New York, New York, NY, United States; ^3^Health Economics and HIV and AIDS Research Division, University of KwaZulu-Natal, Durban, South Africa; ^4^Gender, Sexuality and Health Area, Department of Psychiatry, HIV Center for Clinical and Behavioral Studies, Columbia University Irving Medical Center, New York, NY, United States; ^5^North Star Alliance, Nairobi, Kenya

**Keywords:** HIV, HIV testing, randomized controlled trial, implementation science, HIV self-testing, Kenya, truck drivers

## Abstract

**Background:** Studies suggest that offering HIV self-testing (HIVST) increases short-term HIV testing rates, but few have looked at long-term outcomes.

**Methods:** We conducted a randomized controlled trial (RIDIE 55847d64a454f) on the impact of offering free oral HIVST to 305 truck drivers recruited from two clinics in Kenya. We previously reported that those offered HIVST were more likely to accept testing. Here we report on the 6-month follow-up during which intervention participants could pick-up HIVST kits from eight clinics.

**Results:** There was no difference in HIV testing during 6-month follow-up between participants in the intervention and the standard of care (SOC) arms (OR = 1.0, *p* = 0.877). The most common reasons given for not testing were lack of time (69.6%), low risk (27.2%), fear of knowing HIV status (20.8%), and had tested recently (8.0%). The null association was not modified by having tested at baseline (interaction *p* = 0.613), baseline risk behaviors (number of partners in past 6 months, interaction *p* = 0.881, had transactional sex in past 6 months, interaction *p* = 0.599), nor having spent at least half of the past 30 nights away from home for work (interaction *p* = 0.304). Most participants indicated a preference for the characteristics associated with the SOC [preference for blood-based tests (69.4%), provider-administered testing (74.6%) testing in a clinic (70.1%)]. However, those in the intervention arm were more likely to prefer an oral swab test than those in the SOC (36.6 vs. 24.6%, *p* = 0.029).

**Conclusions:** Offering HIVST kits to truck drivers through a clinic network had little impact on testing rates over the 6-month follow-up when participants had to return to the clinic to access HIVST. Clinic-based distribution of HIVST kits may not address some major barriers to testing, such as lack of time to go to a clinic, fear of knowing one’s status and low risk perception. Preferred HIV testing attributes were consistent with the SOC for most participants, but oral swab preference was higher among those in the intervention arm, who had seen the oral HIVST and had the opportunity to try it. This suggests that preferences may change with exposure to different testing modalities.

## Introduction

HIV self-testing (HIVST) is a new biomedical tool that may facilitate reaching individuals not testing regularly under traditional HIV testing programs. HIVST may address the stigma associated with being seen in a testing clinic as well as privacy and confidentiality (1), especially for groups at high risk for HIV infection and that experience discrimination (2). A 2016 systematic review and meta-analysis found that self-administering and interpreting a rapid HIV test was as accurate as provider-administered testing (2), and in 2016 the World Health Organization (WHO) recommended that HIVST be offered as an additional approach to HIV testing services, rating their recommendation as strong and based on moderate quality evidence (2). A number of randomized controlled trials (RCTs) have found that offering free oral HIVST as an option increases HIV test uptake over the standard of care (SOC) of offering only provider-administered testing (3–15). However, most HIVST studies examined HIV testing rates over a short period of time and there is little evidence that the higher HIV testing rate associated with the initial introduction and availability of HIVST will continue over time. Offering a new product for free may motivate people to try it, but when the initial novelty has worn off, testing rates may revert to baseline. In fact, in one of the trials among Kenyan truck drivers, 89.3% of those who chose to self-test at baseline in the clinic with supervision said that they did so because they were curious to try a new test (16). The follow-up period in most RCTs described to date has been short, no longer than 4 months (4–11). Although three trials among men who have sex with men in the United States, Hong Kong and Australia were somewhat longer, ranging from 6 to 15 months (12–14), they did not look at changes in the intervention impact over time. Thus, decisions about rolling-out HIVST are being based on data from relatively short periods of follow-up, with little evidence that the impact will be sustained over the long-term.

In 2015 we conducted an RCT among 305 Kenyan truck drivers recruited from the waiting rooms of two North Star Alliance roadside wellness clinics. Study participants were all men working as truck drivers with a mean age of 37 years. About 36% had graduated high school and 83% were married. The majority (72%) earned 24,000–55,000 KES per month (about ﹩240–550 US) and had worked as truckers for 8.7 years on average. Ninety-eight percent of participants reported having been sexually active in the past 6 months and 56% had paid for sex during that time period. Participants were randomized to one of two arms in which they were offered (1) a choice between (a) the SOC HIV test (rapid provider-administered finger-prick test in the clinic) or (b) supervised self-administered rapid oral HIVST in the clinic before leaving the clinic (baseline); those who refused both in-clinic options were then offered (c) the HIVST kit to take for use outside of the clinic (i.e., home use) (intervention arm) or (2) the SOC HIV test only (SOC arm). In that study we found significantly higher baseline HIV testing rates among those in the intervention arm than the SOC arm (3).

In this same study, we also informed those in the intervention group that they could access HIVST kits from any of the eight North Star Alliance roadside wellness clinics in Kenya over the following 6-month period. At 6 months post-study enrollment, we interviewed all study participants about HIV testing they had undergone since baseline, as well as preferences regarding future HIV testing, and we report those results here.

## Methods

This RCT was registered prior to initiation in the Registry for International Development Impact Evaluations (RIDIE), ID# 55847d64a454f. The methods have been reported elsewhere (3) but here we provide a brief description. In October–December 2015, we invited all truck drivers who visited two North Star Alliance roadside wellness clinics in Kenya to screen for eligibility for participation in a study on HIV testing. Those who were (1) ≥18 years old, (2) male, (3) worked as a truck driver or trucking assistant, (4) resided in Kenya, (5) spoke English or Kiswahili, (6) self-reported they were HIV-negative or unknown HIV status, (7) were able to sign the consent form, and (8) were willing to receive payment of participation fees via MPesa (a cell-phone-based money transfer system) were eligible to participate. In order to prevent bias, participants were blinded to the study research question and to the fact that they would be randomized to arms offering different HIV testing options. The study was approved by the City University of New York Institutional Review Board, the Kenya Medical Research Institute Ethics Committee, and the University of KwaZulu-Natal Biomedical Research Ethics Committee.

We administered a baseline questionnaire about demographic background, HIV testing history and sexual risk behavior, after which the fieldworker opened a sealed envelope with the randomization assignment. Participants were randomized on a 1:1 basis to either the SOC arm or the intervention arm, stratified by clinic. For those randomized to the SOC arm, the fieldworker offered the standard HIV test, which was a provider-administered rapid finger-prick test conducted in the clinic with pre- and post-test counseling. For those randomized to the intervention arm, the HIVST kit was demonstrated and then they were given a choice between (1) the SOC test or (2) rapid oral HIVST for use in the clinic with provider supervision, and those who refused both in-clinic options were then offered (3) a self-administered oral rapid HIV test kit to take for use outside of the clinic (home use). Those who accepted HIV testing in the clinic underwent standard pre- and post-test counseling procedures while those who took a test kit for home use were given pre-test counseling while in the clinic and post-test counseling by phone after testing. Another questionnaire was administered following testing or test refusal and, before leaving the clinic, those in the intervention arm were informed that they could pick-up HIVST kits from any of the eight North Star Alliance roadside wellness clinics in Kenya over the following 6 months for home use or use in the clinic with supervision, depending on their preference. We contacted all study participants 6 months following study enrollment to ask about HIV testing since baseline, reasons for their testing decisions and preferred HIV testing program attributes for future testing. Participants received the equivalent of approximately ﹩6 US for completing the baseline interview and an additional ﹩4 for completing the 6-month follow-up interview as compensation for their time.

### Statistical Analysis

We previously described the sample overall and compared characteristics by randomization arm. There were no significant differences by randomization arm (3). We calculated Mantel Haenszel odds ratios for HIV testing during the 6-month follow-up period by randomization arm adjusted for clinic (strata used in the randomization scheme). For those in the intervention arm who tested during follow-up, we described what HIV test they used (SOC, HIVST for home use or supervised use in the clinic). For those in both arms who did not test during follow-up, we described the reasons given for not testing and further explored if those reasons might be modifiers of the association found between HIV testing during follow-up and randomization arm using logistic regression with the pertinent 2-way interaction terms and adjusted for clinic. The factors assessed in the interaction analysis were determined *post-hoc*, driven by the factors participants stated as reasons for not testing, and included proxy measures for recent HIV testing (having tested at baseline), HIV risk (number of sex partners and transactional sex in the past 6 months reported at baseline), as well as a proxy for limited free time (report of having spent more than half of the past 30 nights away from home due to work at baseline). Finally, we described the HIV testing program attributes participants reported they would prefer for future HIV testing. All descriptive statistics were examined for the sample overall and then stratified on randomization arm, with a chi square test (or Fisher's exact when expected cell counts were <5) to assess statistical significance. All statistical tests were two-sided at alpha = 0.05 and conducted using SPSS version 25 (Chicago, IL).

## Results

### Study Flow Over 6-Month Follow-Up

**Figure 1:**
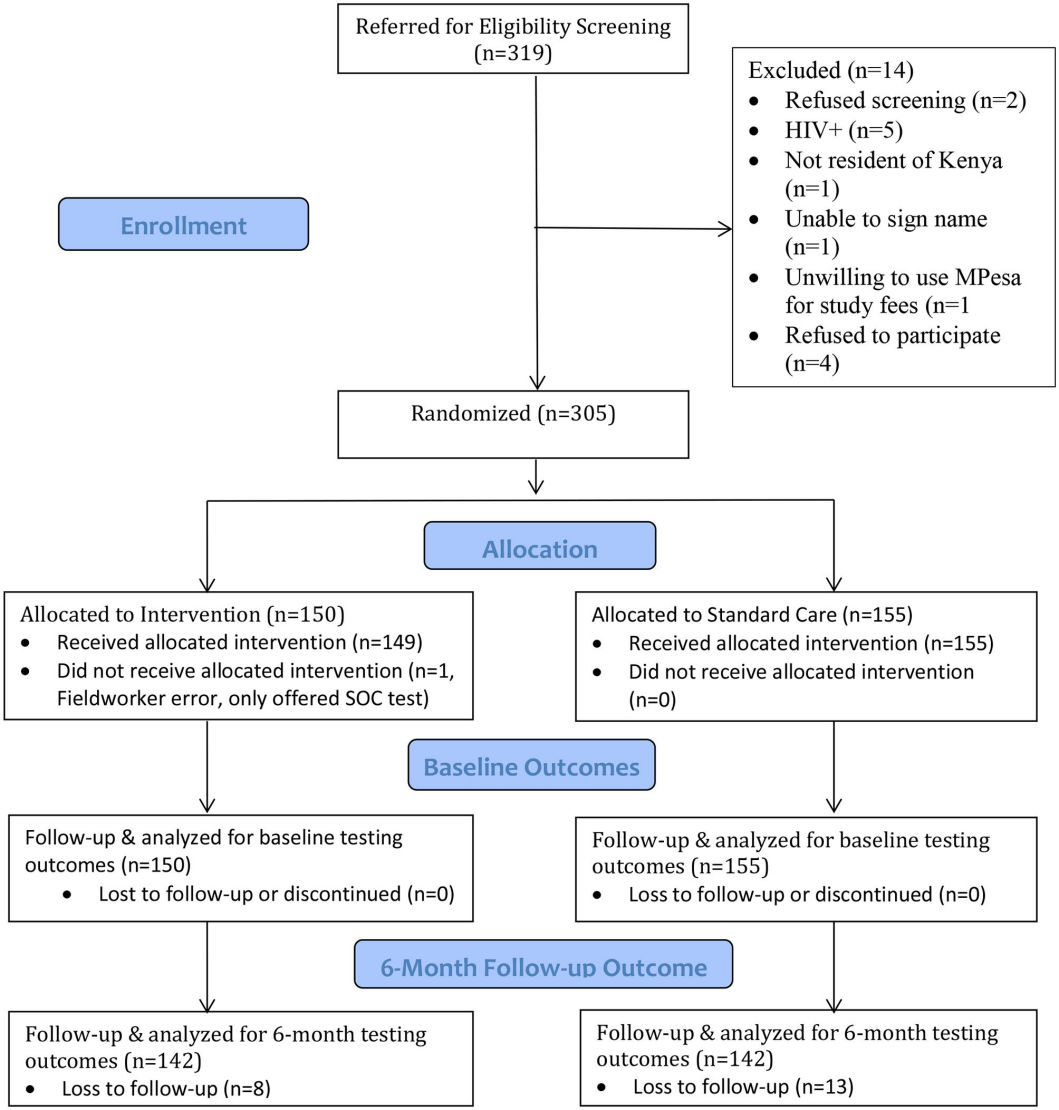
Study flow chart.

The study flow chart is presented in Figure 1. A total of 305 truck drivers were enrolled in the study and completed baseline procedures. Note, one participant in the intervention arm was not offered HIVST as a choice and therefore we analyzed the outcome data both based on intent-to-treat and per protocol. At 6-month follow-up, 21 participants were lost to follow-up (8 in the intervention arm and 13 in the SOC arm), yielding a sample of 284 participants for the 6-month analysis.

### HIV Testing Outcomes Over 6-Month Follow-Up

There was no significant association between randomization arm and HIV testing during the 6-month follow-up in both the intent-to-treat analysis (OR = 1.0, *p* = 0.877) and the per protocol analysis (OR = 0.9, *p* = 0.779) (Table 1). Participants who had not tested for HIV at baseline were more likely to test during the follow-up period (63.4 vs. 54.2%), but the difference was not statistically significant (*p* = 0.236) (Data not shown). The most common reasons given for not testing during follow-up were lack of time (69.6%), perceived low HIV risk (27.2%), fear of test results (20.8%) and having tested recently (8.0%). None of these reasons differed by randomization arm (Table 2). In an attempt to assess whether some of these barriers might be addressed by HIVST, we explored whether the intervention effect was modified by having tested at baseline, a proxy for having tested recently (interaction *p* = 0.613), number of partners in past 6 months reported at baseline (interaction *p* = 0.881) and report at baseline of having had transactional sex in the past 6 months (interaction *p* = 0.599), both proxies for HIV risk perception, and having spent ≥15 of the last 30 nights away from home for work, a proxy for lack of time (interaction *p* = 0.304). None of the interaction terms were statistically significant (Data not shown).

**Table 1:** HIV test uptake overall and by arm under intent-to-treat and per protocol status.

	**Total,** ***n*** **(%)**	**Intervention arm,** ***n*** **(%)**	**SOC arm,** ***n*** **(%)**	**Mantel Haenszel OR (95% CI) adjusting for strata**	**Mantel Haenszel *p*-value**
**Tested at 6 month follow-up (intent-to-treat analysis)**					
Yes	159 (56.0%)	80 (56.3%)	79 (55.6%)	1.0 (0.6–1.5)	0.877
No	125 (44.0%)	62 (43.7%)	63 (44.4%)	NA	NA
Tested at 6 month follow-up (per protocol analysis)^*^					
Yes	159 (56.0%)	80 (56.7%)	79 (55.2%)	0.9 (0.6–1.5)	0.779
No	125 (44.0%)	61 (43.3%)	64 (44.8%)	NA	NA

* *One participant in the intervention arm was only offered the SOC HIV test at baseline, so that individual is analyzed in the SOC arm in the per protocol analysis*.

**Table 2:** Reason for not testing during 6-month follow-up among those who did not test.

	**Total** ***n*** **(%)**	**Intervention,** ***n*** **(%)**	**SOC,** ***n*** **(%)**	***p*** **-value**
Total	125 (100%)	62	63	
Tested recently	10 (8.0%)	3 (4.8%)	7 (11.1%)	0.323^*^
Afraid or don't want to know status	26 (20.8%)	13 (21.0%)	13 (20.6%)	0.963
Not at risk	34 (27.2%)	17 (27.4%)	17 (27.0%)	0.956
Worried about losing job	2 (1.6%)	0 (0.0%)	2 (3.2%)	0.496^*^
Do not trust test results	3 (2.4%)	1 (1.6%)	2 (3.2%)	1.000^*^
Do not trust provider or worried about lack of confidentiality	4 (3.2%)	1 (1.6%)	3 (4.8%)	0.619^*^
No time	87 (69.6%)	42 (67.7%)	45 (71.4%)	0.654
Lack access to HIV care	0 (0%)	0	0	NA

* *Fisher's exact test used*.

### HIV Test Used During 6-Month Follow-Up Among Those in the Intervention Arm

Of the 80 participants in the intervention arm who could access HIVST kits and tested during follow-up, 18 (22.5%) used an HIVST while the other 62 (77.5%) accessed the SOC. Of the participants who self-tested, 3 (16.7%) used the HIVST at the clinic under supervision while the remaining 15 (83.3%) took the kit for home use. Among the 80 participants in the intervention arm who tested during follow-up, those who had self-tested at baseline were more likely to pick up a self-test kit during follow-up (26.1% of those who self-tested in the clinic and 33.3% of those who took a self-test kit for home use at baseline) compared to those who had not self-tested at baseline (20.0% of those who took the SOC test and 7.7% of those who did not test at baseline) but the difference was not statistically significant (Fisher's exact *p* = 0.487) (Data not shown).

### HIV Testing Program Attributes Preferred for Future Testing

When asked about preferences for future HIV testing, the majority of participants selected attributes of the SOC test: blood test (69.4%), provider-administered (74.6%) in the clinic (66.8%). However, 25–30% selected attributes of the HIVST that we made available (30.6% oral swab, 25.4% self-administered and 26.9% at home). Preferences regarding testing alone vs. with a partner were evenly split (47.9% preferred to test alone and 52.1% preferred to test with a partner). The only attribute preference that varied by randomization arm was biological specimen, with a higher proportion preferring the oral swab test among those in the intervention arm (i.e., the group that saw a demonstration of an oral swab HIVST test and had the opportunity to use it) compared to the SOC arm (36.6 vs. 24.6%, *p* = 0.029) (Table 3).

**Table 3:** Preferred attributes of HIV testing programs for future testing.

	**Total,** ***n*** **(%)**	**Intervention,** ***n*** **(%)**	**SOC,** ***n*** **(%)**	***p*** **-value**
**Biological specimen**				
Blood	197 (69.4%)	90 (63.4%)	107 (75.4%)	0.029
Oral swab	87 (30.6%)	52 (36.6%)	35 (24.6%)	
**Administration**				
Provider	211 (74.6%)	100 (70.9%)	111 (78.2%)	0.162
Self	72 (25.4%)	41 (29.1%)	31 (21.8%)	
**Testing alone vs. with partner**				
Alone	136 (47.9%)	64 (45.1%)	72 (50.7%)	0.342
With partner	148 (52.1%)	78 (54.9%)	70 (49.3%)	
**Location**				
Home	76 (26.9%)	35 (24.6%)	41 (29.1%)	0.520^*^
Clinic	201(71.0%)	103 (72.5%)	98 (69.5%)	
Other	6 (2.1%)	4 (2.8%)	6 (2.1%)	

* *Fisher’s exact test calculated in SAS. When excluding the “Other” category, the difference was still not significant (chi-square p = 0.441)*.

## Discussion

When free oral HIVST kits were made available to truck drivers through a clinic network, HIV testing was higher than among those offered only the SOC at baseline when the participants were already in the clinic (3), but it had little impact on testing rates over the 6-month follow-up, when participants had to return to the clinic to access the HIVST. This discrepancy could be attributed to participants having overcome the barrier of presenting at a clinic for HIV testing at baseline, since the participants had been recruited from clinic waiting rooms, whereas over follow-up, that barrier was experienced equally among those in the intervention and SOC arms. A study among female sex workers in Uganda found that offering HIVST kits directly through peers was associated with both a higher initial testing rate and a higher probability of repeat testing over 4-month follow-up compared to making HIVST kits available through healthcare facilities (8). Thus, clinic-based distribution of HIVST kits may not address some major barriers to testing that many face. However, in two subsequent RCTs we conducted in which we sent text messages to truck drivers and female sex workers either reminding them of the availability of general HIV testing at North Star Alliance Clinics (SOC) or announcing the availability of HIVST kits at these clinics, we found higher testing rates among those who received the text messages about the availability of HIVST kits (9, 10). In those studies, participants had to come to the clinic to access both SOC and HIVST testing. It could be that the novelty of making a new product available, in this case HIVST, may be sufficient to overcome the barriers to accessing testing through a clinic, but once the initial novelty has worn off, as may have been the case with the truck drivers in this study who had already been introduced to the HIVST kit and had the opportunity to use it at baseline, the HIVST was no longer sufficiently intriguing to overcome the barriers associated with clinic access. Thus, the impact of new biomedical technology is likely dynamic and uptake may follow a bell-shaped curve rather than the S-shape associated with traditional diffusion theory (17). To put this in the context of health behavior theory, the availability of new biomedical technology might serve as the cue to action in the Health Belief Model, but once that technology is no longer perceived as new, it no longer serves as a cue (18). Of course, this is all conjecture and more research is needed to examine the long-term impact of offering HIVST in general and through different distribution methods outside of the clinic setting.

The reasons given by participants for not testing during follow-up were similar in the intervention and SOC arms. Thus, the primary barriers to self-testing when test kits are distributed for free through clinics and SOC testing appear to be similar and included lack of time, low perception of HIV risk, and fear of the test results. These barriers are likely to directly impact access to HIV testing, be it self-testing or SOC testing, through clinics. Lack of time makes it difficult to fit a clinic visit either for testing or for HIVST kit pick-up into the already busy schedule; lack of risk perception makes adding an inconvenient clinic visit for either testing modality less of a priority; and fear would also make a clinic visit for either testing modality a challenge. Lack of time and low risk perception might be mitigated somewhat through other distribution mechanisms, but fear of an HIV test result requires counseling, a service that is usually accessed once in a clinic. Low risk perception is also something usually addressed through counseling to help people accurately assess their risk and prioritize HIV testing if appropriate. Thus, in addition to considering alternate distribution methods, HIVST programs need to identify mechanisms to address fear of an HIV-positive result and risk perception to increase HIVST uptake. Qualitative interviews with study participants also identified lack of time as an important barrier and an emphasis on the need for counseling (19).

Having tested recently for HIV was the fourth most common reason for not testing during follow-up. The majority of participants in both study arms tested at baseline (72.9% in the SOC and 87.3% in the intervention arm) but high-risk groups in Kenya like truck drivers are counseled to test every 3 months. We attempted to assess if some of the reasons given for not testing over the follow-up period might be mitigated by making HIVST available. We examined these reasons for not testing as possible modifiers of the intervention effect by adding interaction terms to the regression model for having tested at baseline, as a proxy for recent testing, reporting at baseline that ≥15 of the last 30 nights were spent away from home due to work, as a proxy for lack of time, and report at baseline of the number of sex partners and having had transactional sex in the past 6 months, as a proxy for risk, However, none of the interaction terms were statistically significant, suggesting that HIVST distributed through clinics does not address these barriers better than SOC testing.

When participants were asked about their preferences regarding future HIV testing, the majority indicated preference for characteristics of SOC testing (blood-based, provider-administered and in the clinic), but about 25–30% preferred characteristics associated with the HIVST (oral-swab test, self-administered and at home). This suggests that multiple HIV testing options are needed to allow people to access testing modalities that suit their preferences and meet their needs. Interestingly, the proportion of participants who preferred an oral test was higher among those in the intervention group than the SOC group. Since participants were randomized to study arm and therefore confounding is unlikely, although not impossible, this may indicate that having seen a demonstration of the oral swab test and had the opportunity to try it made it more acceptable and even preferred by more people. Thus, HIV testing preferences may change over time, especially with greater knowledge and experience with HIVST.

This study had a number of limitations that should be considered when interpreting the results. First, we had some loss to follow-up (7%), which could have biased our results and reduced statistical power. Furthermore, our assessment of effect modification was a *post-hoc* analysis to try to understand the null results for the impact of our intervention on HIV testing over follow-up. *Post-hoc* analyses looking at effect modification can result in small numbers within certain strata and tend to be underpowered. This may have been the case in our *post-hoc* assessment of possible effect modifiers, and the null results should be viewed with caution. In addition, social desirability bias may have affected how some participants responded to our questions, especially regarding the HIV testing outcome, which may have been over-reported by participants in both arms. The HIV testing rate among study participants in both study arms at baseline was much higher than the 60% testing rate at North Star Alliance clinics during the same time period (3). This may also indicate that our sample was not representative of North Star Alliance roadside wellness clinic clients in general and certainly our results cannot be generalized to all truck drivers in Kenya, let alone other countries.

Despite these limitations, this is one of the first studies that looks at both the short- and long-term impact of the availability of HIVST on HIV testing rates. While making HIVST available to various population groups and using different distribution methods has been found to increase HIV testing rates (3–15), the short study duration makes it hard to determine what the long-term impact might be when HIVST is rolled-out on a wider scale. In this study, our short-term HIV testing outcome was consistent with other studies in finding higher testing rates when HIVST was offered, but the lack of a difference over the 6-month follow-up period leads to concerns that the short-term intervention effect found in most studies may wane over time. This needs to be better evaluated before HIVST programs can be designed to maximize their impact on the HIV epidemic.

## Data Availability Statement

The datasets for this study can be found in the Harvard Dataverse repository (https://dataverse.harvard.edu/dataset.xhtml?persistentId=doi:10.7910/DVN/8GVXJY).

## Ethics Statement

The studies involving human participants were reviewed and approved by City University of New York Institutional Review Board, the Kenya Medical Research Institute Ethics Committee, and the University of KwaZulu-Natal Biomedical Research Ethics Committee. The patients/participants provided their written informed consent to participate in this study.

## Author Contributions

EAK led the conception and design of the study and conducted the data analysis and drafted this manuscript. GG, JEM, EM, and KG were all major contributors to the study design and coordination and participated in data interpretation and manuscript revision. MLR was responsible for data management and study monitoring and participated in data interpretation and manuscript revision. ENN and JOO were responsible for day-to-day study management and oversight and participated in data interpretation and manuscript revisions. All authors read and approved the final manuscript.

## Funding

This study was supported by a grant from the International Initiative for Impact Evaluation (3IE# TW2.2.06, EAK, Principal Investigator). EAK was also supported by Einstein-Rockefeller-CUNY Center for AIDS Research (P30-AI124414) which was supported by the following National Institutes of Health (NIH) Co-Funding and Participating Institutes and Centers: NIAID, NCI, NICHD, NHBL, NIDA, NIMH, NIA, FIC, and OAR. Support for JEM also came from a center grant from the National Institute of Mental Health (NIMH) to the HIV Center for Clinical and Behavioral Studies at the New York State Psychiatric Institute and Columbia University Irving Medical Center [P30-MH43520].

## Author Disclaimer

The views expressed in this article are not necessarily those of 3IE or its members.

## Conflict of Interest

The authors declare that the research was conducted in the absence of any commercial or financial relationships that could be construed as a potential conflict of interest.

## Publisher's Note

All claims expressed in this article are solely those of the authors and do not necessarily represent those of their affiliated organizations, or those of the publisher, the editors and the reviewers. Any product that may be evaluated in this article, or claim that may be made by its manufacturer, is not guaranteed or endorsed by the publisher.
